# Disruption of State Estimation in the Human Lateral Cerebellum

**DOI:** 10.1371/journal.pbio.0050316

**Published:** 2007-11-27

**Authors:** R. Chris Miall, Lars O. D Christensen, Owen Cain, James Stanley

**Affiliations:** 1 School of Psychology, University of Birmingham, Birmingham, United Kingdom; 2 Department of Experimental Psychology, University of Oxford, Oxford, United Kingdom; University of Minnesota, United States of America

## Abstract

The cerebellum has been proposed to be a crucial component in the state estimation process that combines information from motor efferent and sensory afferent signals to produce a representation of the current state of the motor system. Such a state estimate of the moving human arm would be expected to be used when the arm is rapidly and skillfully reaching to a target. We now report the effects of transcranial magnetic stimulation (TMS) over the ipsilateral cerebellum as healthy humans were made to interrupt a slow voluntary movement to rapidly reach towards a visually defined target. Errors in the initial direction and in the final finger position of this reach-to-target movement were significantly higher for cerebellar stimulation than they were in control conditions. The average directional errors in the cerebellar TMS condition were consistent with the reaching movements being planned and initiated from an estimated hand position that was 138 ms out of date. We suggest that these results demonstrate that the cerebellum is responsible for estimating the hand position over this time interval and that TMS disrupts this state estimate.

## Author Summary

Motor control depends on the brain's awareness of the current state of the body. Knowing the current position and movement of the arm, for example, allows one to reach rapidly and accurately towards a target. However, sensory information reaches the brain only after a short delay, and the arm may already be in motion. Therefore, it has been proposed that the brain must calculate a “state estimate”—by combining sensory information about the last known position of the arm with predictions of its responses to recent movement commands—which it uses to accurately plan and control a reaching movement. To test this idea, we used transcranial magnetic stimulation to briefly disrupt several separate areas in the brain as participants reached to a target. We show that stimulation over the cerebellum caused reaching errors consistent with movements planned on the arm's position about 140 ms previously, whereas stimulation of other brain areas did not disrupt reaching direction. These results add weight to the hypothesis that the cerebellum predicts the state of the motor system. This hypothesis can explain the loss of movement control experienced by cerebellar patients and supports computational theories that the cerebellum is a predictive model of the motor system.

## Abstract 3

Transcranial magnetic stimulation of the human cerebellum causes errors in reaching movements that are consistent with a temporary disruption in estimating the arm's current state.

## Introduction

The central nervous system (CNS) can never know exactly the current state of the peripheral motor apparatus—the limbs and muscles that are under CNS control—because of unavoidable delays in conduction of sensory afferent signals from the periphery, as well as in their central neural processing. Hence the sensed state of the system (the set of variables including limb segment positions and velocities that capture its behaviour) always lags behind its true state [1]. These delays vary with the sensory modality but can be substantial, and estimates of the delay involved in using visual feedback to control and correct ongoing movements vary from about 100–300 ms [2–5]. In addition, any physiological sensor will have some inaccuracies, compounded by neural noise, that lead to errors in the measurements. Furthermore, the parameters that the CNS might aim to control, such as the position or velocity of the peripheral motor system, are often hidden from the CNS by indirect relationships between these peripheral variables (muscle lengths or joint angles) and the sensory encoders. For example, vertebrate joint angles are encoded mainly in information carried by muscle spindles, which can only provide a mixed signal that is proportional to muscle length and its rate of change. To measure and control the kinematics of a movement requires decoding these afferent signals to estimate joint angles from muscle lengths. In addition, combining an independent prediction of the state of the peripheral apparatus with afferent measurements of its state can provide an estimate that is more accurate than that of either predictor or sensors alone [6]. For these various reasons, it is widely assumed that the brain generates an estimate of the true state of the peripheral motor system, by integration of the latest afferent sensory information with an efferent copy of motor commands using prior knowledge of the relationships between efferent signals and the subsequent sensory reafference [7–10]. The process of translating an efferent copy of a motor command into predicted sensory reafference is encapsulated by the idea of a forward model [7,11]. A forward model receives efferent copies of the motor commands and also receives sensory inputs that describe the motor state. The output of the model is a prediction of the sensory consequences of the motor command, i.e., a prediction of the change in motor state. State estimation must be a predictive process because of central delays in processing of the motor command, in peripheral conduction of the efferent signal, and in neuromuscular excitation-contraction coupling. Hence the true motor state of the motor periphery lags behind the central (CNS) changes in motor commands. The state estimation process is therefore inseparably coupled to the process of forward modelling [11].

Forward modelling has been proposed to be a key function of the cerebellum [7,12,13], and the cerebellum has been specifically linked to state estimation [14,15], possibly in conjunction with the superior parietal cortex [4,16–18]. The cerebellum receives appropriate ascending proprioceptive inputs and the efferent copies of descending motor commands, and it outputs to cortical and brain stem motor nuclei [12]. It also has the necessary adaptive mechanisms to support this hypothesised role, because the forward model predictions must be refined and maintained by experience-based motor learning [11,12]. However, to date, there has been no direct experimental evidence of this cerebellar contribution to state estimation; indirect evidence has been derived from brain imaging [19–22] and from studies of cerebellar patients with chronic lesions [23–25].

A loss of state estimation would lead to inaccuracies in motor control, because control signals would be based on out-of-date information. Thus a rapid reaching action made without state estimation of the moving hand would tend to overshoot its target, because information that the desired target had been reached would only arrive at the CNS after the hand had passed beyond. This would result in movement errors analogous to the hypometria of cerebellar patients [12]. State estimation is also important for the synchronous and coordinated activation of different motor effectors. If the future state of one effector can be predicted, then control signals to the other can be issued to produce simultaneous actions, which are a key feature of coordinated action. Without these predictions, the two effectors could only be controlled reactively [23,24], after measurement of the outcome of each command. The loss of coordination and asynchrony of joint actions that would be expected from a failure of state estimation are again similar to the poorly coordinated and ataxic movements of cerebellar subjects [12,26,27]. Thus, there is theoretical and experimental evidence to suggest that the cerebellum is involved in state estimation. To date, we are aware of no studies that have directly tested this hypothesis by experimental disruption of the cerebellum.

So, to further test the hypothesis that the human cerebellum is involved in the generation of a state estimate, we have used transcranial magnetic stimulation (TMS) over the ipsilateral cerebellum during voluntary arm actions to briefly disturb its function. We used a task in which humans were required to make a slow, lateral, but untargeted movement with their arm before being suddenly cued to make a rapid pointing movement towards a static target. Accurate reaching in these circumstances requires up-to-date knowledge of the arm's moving position at the moment of the go cue. Any failure to estimate the arm's initial state caused by the cerebellar TMS should be evident as inaccurate movement. However, because of its location, the human cerebellum is difficult to stimulate with transcranial coils, and TMS targeted at the lateral cerebellum can also directly stimulate neck muscles, the brachial plexus, muscles in the neck or shoulder, and is sufficiently loud that it can provide a startling stimulus affecting speed of movement onset. We have used a series of control conditions to separate nonspecific effects from a specific change in initial movement direction and in terminal error, which were seen only with cerebellar TMS.

## Results

**Figure 1:**
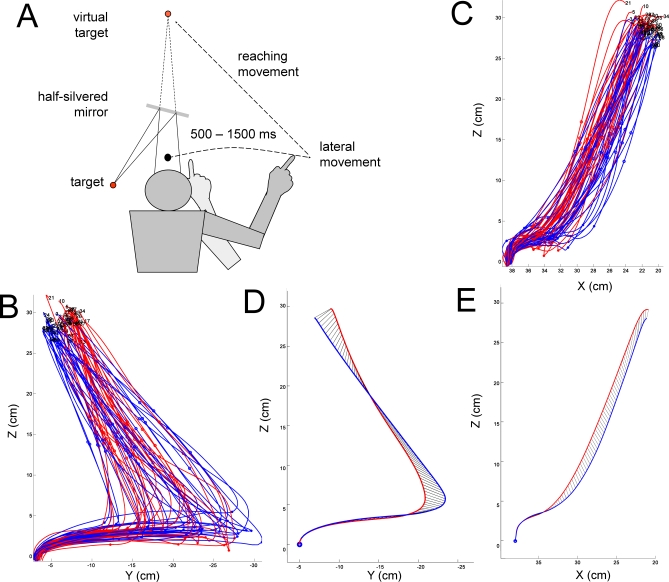
The Experimental Task and Typical Single-Participant Data The experimental task (A), individual trial data (B and C), and session averaged data (D and E) (*n* = 30 trials) from one typical participant as TMS were applied over the right lateral cerebellum. (B and D) show the finger trajectory viewed from behind and from the right of the subject (C and E). In all panels, TMS trials are plotted in red and non-TMS trials are in blue. Clockwise rotation in (B and D) is defined as increasing azimuth angle; clockwise rotation in (C and E) is defined as decreasing elevation angle.

Participants viewed a virtual image of a static target in three-dimensional (3-D) space ahead of them, and started each trial by lifting their right index finger from a start key and moving steadily towards their right (Figure 1A). Liquid crystal device (LCD) goggles blocked the view of their hand and of the target as soon at the start key was released. An auditory go cue, 500-1500 ms after trial onset, instructed them to make a rapid upwards- and leftwards- pointing movement to the virtual target. Their index finger had typically moved laterally 10–40 cm from its original position when the go cue was delivered (Figure 1B). Final positional errors on control trials were small (Figure 1B) and averaged 4.2 cm across all conditions. Thus, participants were normally able to compensate for their initial lateral arm motion and reach the target despite the lack of visual feedback. Vision was allowed after the reach-to-target motion was complete, avoiding any slow drifting of accuracy across trials. However, on a random 50% of trials in each block, TMS was delivered within their reaction time after the auditory go cue, in order to disrupt the planning and initiation of the reach-to-target movement. Reaction times for control trials without TMS averaged 265 ms (discussed later), but were reduced to 170 ms during TMS trials; the three TMS pulses were delivered at 50, 100, and 150 ms during this interval.

### Cerebellar TMS Increases Final Error

The short train of three TMS pulses delivered over the lateral cerebellum caused a significant within-subject increase in mean error for TMS trials compared with non-TMS trials. In our initial experiments, we tested eight participants with TMS over the right ipsilateral cerebellum, the contralateral (left) motor cortex, and the ipsilateral neck, using separate recording sessions separated by at least one day. The mean increase in end-point errors with the cerebellar stimulation site was 36% (2.26 cm ± 0.37 standard error of the mean [SEM], *n* = 8, *t*(7) = 5.72, *p* < 0.0001), and was significantly higher than the other two conditions (repeated measures analysis of variance [ANOVA], *F*(2,14) = 4.468, *p* = 0.032).

However, this reduction in pointing accuracy could have been a nonspecific effect of the TMS stimulation, which can be uncomfortable and even startling. To include other control conditions, we then expanded our cerebellar test group to a total of 32 participants, testing each participant in this main condition of interest as well as in one or more other conditions. Because the extra participants were not tested in all other stimulation conditions, the following analyses are reported as between-group comparisons.

**Figure 2:**
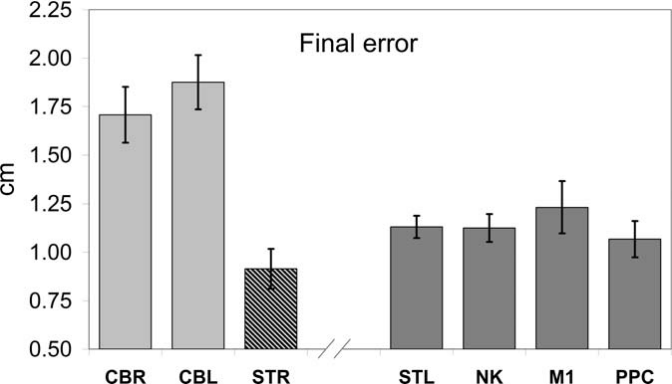
TMS-Induced Difference in Mean End-Point Error Each bar is the group mean difference for TMS versus non-TMS trials (+1 SEM). TMS was applied over the cerebellum during rightwards and leftwards movement (CBR, *n* = 32, CBL, *n* = 13) and when stationary (STR, *n* = 9). Control conditions included during startle trials (STL, *n* = 11), stimulation of the ipsilateral neck (NK, *n* = 10), the hand area of contralateral primary motor cortex (M1, *n* = 20), and the contralateral posterior parietal cortex (PPC, *n* = 12).

With this expanded dataset (Figure 2A), the increase in mean terminal errors in cerebellar TMS trials compared to non-TMS trials was reduced from 36% to 23.7% (or 1.71 cm ± 0.144 SEM, *n* = 32, *t*(31) = 3.80, *p* < 0.001). However, this TMS-induced error was still significantly higher when compared with stimulation lower on the neck (1.12 cm, *n* = 11), or over the hotspot in the primary motor cortex for inducing visible twitches in the first dorsal interosseous muscle in the hand (1.23 cm, *n* = 21), and higher than when startling auditory clicks were presented either using the TMS coil over the ear or using ear phones without TMS (1.18 cm, *n* = 4 and 7, respectively). It was also higher than with stimulation over the contralateral posterior parietal cortex (1.07 cm, *n* = 12), which was targeted using the coordinates of the P3 electrode in the 10–20 electroencephalogram (EEG) electrode positioning scheme [28]. A one-way ANOVA with five conditions (cerebellum, neck, startle, parietal, and motor cortical stimulation) was significant (*F*(4,78) = 3.79, *p* = 0.007, and post-hoc comparisons of the cerebellar condition with the other four conditions were all significant, *p* < 0.025). The difference from stimulation over the hand area of the contralateral motor cortex was smallest (*p* = 0.025); the other four control conditions were not significantly separable from each other (*p* > 0.27)]. Thus, whereas each of these sites induced some increase in end-point error, presumably due to the nonspecific effects of the stimulation, the effects caused by stimulation over the ipsilateral cerebellum were most pronounced and statistically reliable

**Figure 3:**
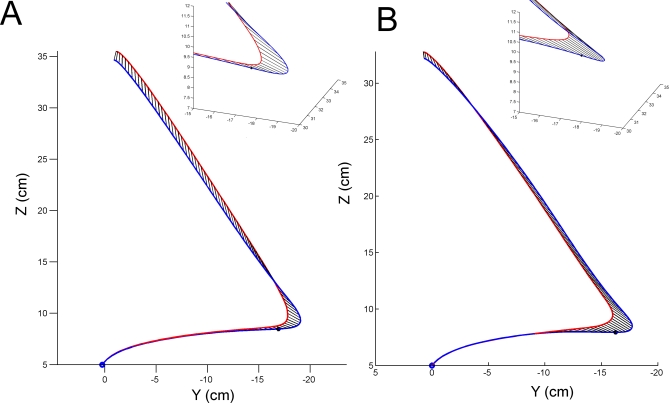
Group Mean Trajectories Group mean trajectories (A) for TMS trials (red) and non-TMS trials (blue) applied over the cerebellum (*n* = 32). (B) Results from startling TMS or auditory trials, without cerebellar disruption (*n* = 11). In both panels, the curved path followed from bottom left to right is during the pre-cue period. Shortly after the go cue and TMS, a rapid reach-to-target towards the upper left target position is made. The 3-D inset figures show an expanded view of the reach-to-target initiation. Black dots mark the position on the non-TMS mean trajectory (blue line) from which a similar angular deviation between start and maximum velocity would be found as seen in the TMS trials.

The increase in error was partly due to a 14% increase in end-point variability across trials. However, the RMS end-point standard deviation measured across all three dimensions was not significantly different for any of the five conditions (*p* > 0.103). There was also a significant end-point positional bias for cerebellar stimulation, as the TMS trials ended on average 1.0 cm above and slightly behind the non-TMS trials (Figure 3A). This corresponds with a small hypermetric overshoot and a small directional error; the increase in overall amplitude of the reach-to-target movement did not reach statistical significance (*p* = 0.14).

### Baseline Performance Differences

Our analysis compares within-subject average errors across sessions including 30 TMS and 30 non-TMS trials. To estimate the expected level of difference between these means due to random sampling from a distribution of variable movements, we analysed the training data for 18 participants before TMS was applied. In training, the TMS machine was placed behind the participant and was triggered exactly as in the test sessions, so that its activation was audible to the participants but had no direct effect. Trials were then grouped by TMS activation versus nonactivation. As expected, there were no significant differences between movement trajectories, reaction times, or peak velocities of the movements. The mean terminal errors differed by 0.40 cm (±0.1 cm SEM, *n* = 18) and the average spatial separation of the mean end positions in the two data sets was 0.8 cm (±0.09 cm SEM, *n* = 18). This suggests that random sampling of any one of our datasets would produce differences representing about half of that seen in our control conditions (Figure 2), and less than a quarter of the effect seen for TMS over the cerebellum. Moreover, these figures (50% and 25%) are conservative, based on 18 training sets compared with the total sample of 32 for the cerebellum and about 10–12 for other conditions. The larger datasets would be less affected by random sampling.

### TMS Effect Is Exposed by Dynamic State Change

TMS took place during the reaction time between the go cue and the start of the reach-to-target movement, while the hand was being actively moved towards the right. Thus, the TMS-induced error for the cerebellar stimulation condition is, we hypothesize, due to the disruption of the state estimation process within the ipsilateral cerebellum and would affect the estimation during the current rightwards movement. If true, then the effect should not be seen if the need for state estimation was minimized. We therefore compared TMS stimulation at the same cerebellar location but with the participant holding their arm stationary at the moment of cue onset. In this control condition, the starting button was shifted 20 cm laterally, to be coincident with the mean start position of the hand in other conditions, and the participant was instructed to lift the finger from the start button but to then remain stationary until the auditory go cue. Hence the starting position was known and static prior to cue onset. A reach-to-target from this fixed position would not require renewed state-estimation because the state was constant and up to date throughout the reaction time period. End point errors were significantly lower in this condition than with cerebellar stimulation (0.92 cm, *n* = 9) and were not significantly different from the other control conditions (Figure 2). Hence the TMS-induced effect is specific to those conditions in which the initial state of the arm is dynamically changing, when its true state must be estimated,

### Cerebellar TMS Causes Initial Aiming Error

The duration of the cued reach-to-target movement was about 725 ms (723.5 ms with TMS over the cerebellum, 725.9 ms without), allowing time for an initial error in the onset of the reach to be corrected during its execution. Hence, although they are significant, the final errors reported above may only reflect a small part of the disruption caused by the TMS. We therefore measured the angular deviation in the initiation of the reach-to-target movement. Individual trials started from different positions (Figure 1B), so we measured for each trial the angular difference between two lines—one joining target position to the hand position at the start of the reach-to-target, and one joining the starting hand position to its position at maximum velocity—and we compared this angle within participants across TMS versus non-TMS trials (see Methods). Because the hand was travelling in a predominantly rightwards direction at cue onset (with the average speed in that direction 760% greater than upwards, and 490% greater than forwards), we expect the errors to be most prominent in azimuth angle (Figure 1B)

**Figure 4:**
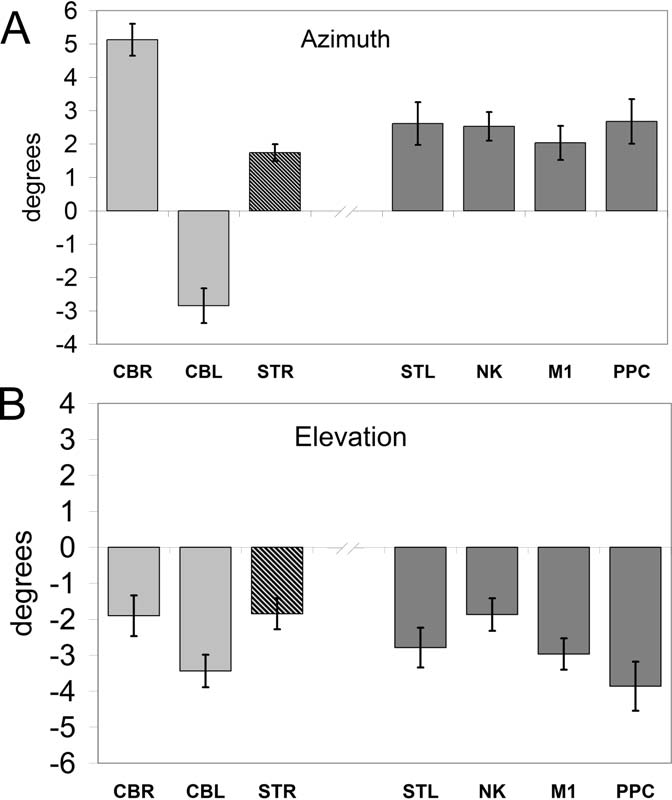
TMS-Induced Difference in Mean Azimuth (A) and Elevation Angles (B) Each bar is the group mean difference for TMS versus non-TMS trials (+1 SEM); see Figure 2. (A) Positive azimuth angles are defined as clockwise rotations in the frontal plane (see Figure 1B and 1D). (B) Negative elevation angles are defined as clockwise rotations in the sagittal plane (Figure 1C and 1E).

For stimulation over the cerebellum, the 5.13° (±0.48° SEM) difference in azimuth was highly significant (Figure 4A, one-sample *t*-test, *t*(32)=10.58, *p* < 0.0001). The azimuth angle differences between groups were also highly significant [one-way ANOVA (*F*(4,78) = 5.43, *p* = 0.001; post-hoc LSD *t*-test comparisons showed the cerebellar stimulation condition differed from all others (*p* < 0.013); the other four control conditions were not significantly separable from each other (*p* > 0.45)]. In all five conditions, the changes in elevation angle between stimulated and normal trials were not significantly different from each other [Figure 4B, one-way ANOVA (*F*(4,78) = 1.11, *p* = 0.36; post-hoc LSD *t*-test comparisons showed no significant differences (*p* > 0.22), except between cerebellar and parietal stimulation, which approached significance (*p* = 0.06, uncorrected)]. Hence TMS over the cerebellum induced a significant change in the initial direction of the targeted reach, which was partly but not fully corrected by the end of the movement (Figure 3A).

Testing the differences in the training datasets, where TMS was distant from the head and thus ineffective, we found that the mean azimuth angle differences between the two sets of trials was 0.3°, which is approximately 6% of the difference recorded for cerebellar TMS, and not significantly different from zero (one-sample *t*-tests, *p* = 0.7, *n* = 18).

### Direction Specificity

**Figure 5:**
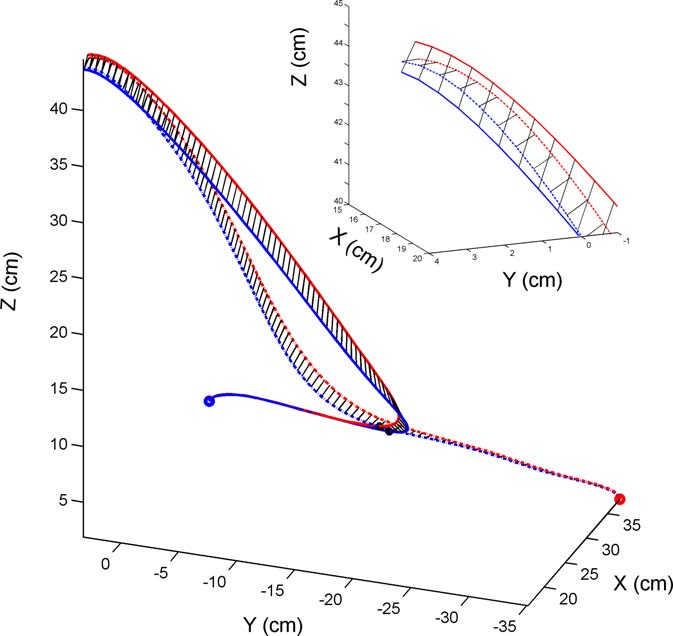
Group Mean Trajectories for TMS Trials (Red) and Non-TMS Trials (Blue) Applied over the Cerebellum (*n* = 13) Solid lines indicate stimulation during initial rightwards movement; dotted lines show stimulation during initial leftwards movement. The deviation between between TMS and non-TMS trajectories at the start of the reach towards the final target is reversed between the two conditions, while final errors are similar. The insert at top right is the terminal portion of the trajectories, rotated into the frontal plane. This emphasises the greater overshoot in the *z-*axis for rightwards TMS trials (red solid lines) compared to leftwards TMS trials (red dotted lines), which mainly overshot in depth (*x*-axis).

The direction of the initial pointing error (a clockwise deviation relative to non-TMS trials) suggests that the reaching movement towards the target was inaccurately planned. We distinguish two possible reasons. One is that there could be a direction-specific effect due to mislocation of the arm at the initiation of the targeted reach, during the rightwards movement from the start key. If the reach-to-target movement was planned based on out-of-date information, i.e., on an estimated start position leftwards of the actual position of the hand, then the movement direction would be rotated clockwise. To test this hypothesis more directly, we include one additional group of participants, who were tested with TMS over the cerebellum during movements made from a far-right position, such that the arm at the go cue was, on average, at the same position, but was moving leftwards (Figure 5). This group showed end-point error amplitudes (mean 1.88 cm, *n* = 13) inseparable from the original cerebellar group (1.71 cm, *n* = 32), and as before, significantly greater than all the other control conditions (Figure 2, one-way ANOVA, *F*(5,69) = 3.57, *p* = 0.006, post-hoc *t*-tests *p* < 0.005). The mean azimuth angle was −2.84°, in other words, rotated counterclockwise, and hence statistically different from all other conditions (Figure 4A, *p* < 0.025); the mean elevation angle did not differ from other conditions (Figure 4B, *p* > 0.19).

The second possibility is that the TMS caused the participants to mislocate the target, and they were therefore accurately reaching to the wrong location. We can dismiss this with the stationary start position data. In this case, there was very little azimuth error (1.74°, not significantly different from all other control conditions), and the end point errors were actually the smallest observed. In addition, we correlated the directional errors in azimuth and elevation for both groups (cerebellar TMS during rightwards and leftwards movements) against the end-point errors in the *x-*, *y-*, and *z-*axis (Figure 1). We hypothesize that if TMS caused target mislocation, then the initial angular deviations would be correlated with the final positional errors. However, we found no evidence to support this: there was only one near-significant correlation between change in azimuth angle and change in *x* error during leftwards movement condition (*p* = 0.061, Bonferroni adjusted); this is not the axis in which changes in azimuth angle would be most prominent, given the near-vertical plane of movements (Figure 1).

Thus we interpret this as further evidence that the initial direction was inaccurate and that some but not all of this error was corrected during the reach. We find no evidence that the final position was mislocated and that the initial angles were altered to reach this final location.

### Estimating the Internal Error in Hand State

To estimate the hand state used to plan the reach-to-target action, we could backtrack along the mean trajectory of non-TMS trials, participant by participant, to find a point at which the angle towards the maximum velocity position was equal to the mean angular deviation seen for that participant in TMS trials. In other words, by assuming that the angular error in aiming to the target was due to a failure of the cerebellar state estimation of the hand position, during the initial slow movement, we found that the prior position of the hand—before the go cue—at which the angular difference between start and maximum velocity points would be the same as was found between TMS and non-TMS trials. This position was 3.97 cm (± 1.01 SEM) leftwards of the actual reach-to-target start position (black dot, Figure 3A). Converting from positional differences along their mean path between TMS and non-TMS trials, and given the mean hand speed at the cue onset, measured subject-by-subject, the distance of 3.97 cm suggests that the reach-to-target was planned based on the hand's position 138 ms (±19 ms SEM) previously. This “state-estimation interval” is significantly greater than zero (one-sample *t*-test, *t*(18) = 7.33, *p* < 0.0001) and is also greater than the change in reaction time caused by the TMS (one-tailed paired-sample *t*-test, *t*(18) = 2.011, *p* = 0.030; see “nonspecific effects” below). For the group tested making initially leftwards arm movement, the “state-estimation interval” was almost exactly the same, 134 ms (±17 ms SEM). This prediction interval cannot be calculated for the stationary-start condition, because backtracking on a stationary trajectory is not possible.

### Other Nonspecific Stimulation Effects

**Figure 6:**
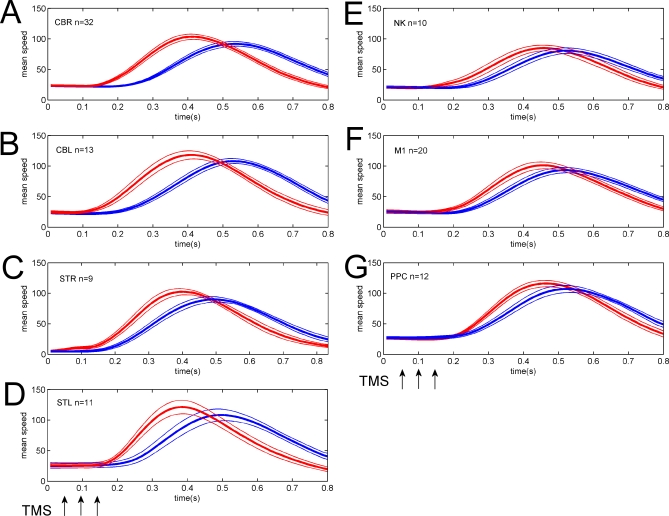
Group Speed Profiles for TMS Trials (Red) and Non-TMS Trials (Blue) Each panel shows the group average speed profile (±1SEM) for 800 ms after cue onset. The time of TMS stimulation is indicated by the three arrows. (A–D) Startle effects: TMS over cerebellum during rightward movement (A: *n* = 32); during leftward movement (B: *n* = 13), or with a stationary start position (C: note zero intial velocity; *n* = 11) leads to a reduced reaction time and increased peak speed very similar to that induced by startle stimulation (D: with TMS over the ear, *n* = 4, or with sound stimuli, *n* = 7). (E–G) Control stimulation sites with TMS over the ipsilateral neck (E: *n* = 10), contralateral hand area of motor cortex (F: *n* = 20) or contralateral posterior parietal cortex (G: *n* = 12); the startle effects are smaller with slighter reduction of reaction time and increase of peak speed.

The velocity profiles of the reach-to-target movements were significantly altered by cerebellar TMS (Figure 6A–6C). The effect was to reduce the reaction time for the rapid reach-to-target movement by about 80 ms and to increase the peak velocity by about 15%. For the main condition of interest, with TMS over the lateral cerebellum during ongoing movement, the mean reaction time for TMS versus non-TMS trials was 172.5 ms versus 265.9 ms. TMS-induced startle effects have been reported previously [29] because of the noise and cutaneous stimulation. TMS over the cerebellum using the large double-cone coils does cause noticeable auditory stimulation, as well as cutaneous and muscular stimulation. Very similar effects on the movement profiles were seen in a control group (Figure 6D) tested with TMS stimulation over the right ear (*n* = 4) or with startling auditory white noise bursts played over headphones (*n* = 7), confirming that the reduction in reaction time and increase in peak velocity is likely to be due to a startle response. For this startle control group (*n* = 11), the mean reaction time was 175.2 ms for startle trials, and 260.7 ms for nonstartle trials. Considerably weaker effects were seen for TMS stimulation over the neck (Figure 6E), the motor cortex (Figure 6F), or posterior parietal cortex (Figure 6G). The reduced reaction time seen for motor cortical TMS has also seen in other TMS experiments [30,31] and is attributed to an alerting but nonstartling effect of the TMS stimulus.

Time to maximum velocity after the onset of the reach-to-target movement was similar (247 ms versus 258 ms for cerebellar TMS versus non-TMS trials and 227 ms versus 250 ms for startle versus nonstartle trials). Overall movement duration was nonsignificantly shorter in the TMS or startle trials. For the cerebellar group, mean duration was 723 ms for TMS trials and 726 ms for non-TMS trials. For the startle group, mean duration was 693 ms versus 726 ms. Thus, there was a subtle increase in the deceleration phase of the movement.

## Discussion

We have shown that a brief train of TMS over the lateral cerebellum, applied during the reaction time to initiate a rapid reaching movement towards a remembered target, resulted in a directional deviation of the reaching movement and in increased positional error. We suggest that the TMS temporarily blocked the contribution of the lateral cerebellum to state estimation. As a result, the reaching movements were planned based on the residual, out-of-date knowledge of the previous state of the arm. Thus, we propose that during rightwards arm movements the arm was estimated to be further leftwards than its true position, while during leftwards movement it was estimated to be further rightwards. These errors lead to clockwise and counterclockwise directional deviation of the initial movement towards the target. When the arm was stationary at movement onset and hence the state estimate was unchanging, then the direction of the reaching movements was largely unaffected by the stimulation.

Our results therefore provide further evidence for a contribution by the cerebellum to state estimation and, by inference, to forward modelling the sensory consequences of action [12,22,32,33]. These forward model predictions, combined with independent sensory information from the periphery, provide an optimal estimate of the current state of the arm. However, there is considerable debate about the role of the cerebellum in motor control [14,20,34,35] and other functions, such as timing, motor learning, predictive control, and inverse dynamic modelling, have all been proposed. We believe the present data are best understood in the context of state estimation. First, predictive control [36,37] is very closely related to state estimation, because the consequences of motor commands must be predicted in order to update and control subsequent actions. Hence state estimation is a subset of predictive control [1]. Next, the cerebellum appears important in timing, especially during discrete motor tasks [38,39]. The timing component of our task is minimal as movements were initiated after a random interval, eliminating a timing strategy and participants were instructed to reach as fast as possible after the go cue. Furthermore, the target was static, and so the directional differences in reaching behaviour in TMS versus non-TMS trials are difficult to justify by timing alone.

Another important postulated function is in inverse dynamic modelling, with a functional role for the cerebellum in generating motor commands rather than in predicting their outcome [7,33,40]. Again, the pattern of results we have shown is not easily fit by this hypothesis. In particular, the reach-to-target from a stationary position was not different from control conditions, whereas disruption of an inverse model would affect all movement for which the model was used. These data also argue against an explanation that the TMS impaired control of interjoint dynamics. Failure to compensate for interjoint dynamics, which has been proposed as an explanation for cerebellar ataxia [27], might contribute to our results, as limb dynamics would be different for the movements made from left and right. However, the initial movements were relatively slow (mean velocity 27.9 cm/s, Figure 6) and so these dynamic effects would be small in comparison to their effects during the much faster reach-to-target action. Indeed, the reach-to-target made from the static position was as rapid (peak velocities of about 100 cm/s, Figure 6C) as in other conditions and, we assume, was fast enough to expose weak interjoint coordination. Its trajectory was not different from the control conditions. But whether these effects contribute more subtly to our overall results will require further work, perhaps using faster baseline movements so that both the dynamic effects and the positional misestimation are larger.

It is also thought that state estimation is required to minimize timing differences between effectors during coordinated actions [41,42]. Loss of coordination is one of the cardinal symptoms of cerebellar dysfunction [43] and is evident as gross ataxia as well as in more subtle measures such as the failure to coordinate grip and lift forces during object manipulation [23,24,44]. Recently, Deidrichsen et al. tested coordination of a reach and a button press and were able to separate experimentally time-dependent and state-dependent behavioural strategies. They found that functional activation changes in the lateral cerebellum were better explained by state estimation than by timing [45], consistent with the present results.

Our estimates of the angular deviations imply that the rapid reaching movements were planned on information that is about 138 ms out of date, an interval that is in the appropriate order of magnitude of sensory reafference [2,3,5]. From this result, we would predict that cerebellar pathology would lead to movement control based on the arm's state about 138 ms less advanced than actual. This would result in direction- and speed-specific deviations in the initial segment of any rapid movement, especially those made during ongoing action, as we have shown here. It would also lead to hypermetria, as the state estimate would be disturbed throughout the reaching movement, rather than just at its initiation. Thus, even the final stages of reaching to a target would be affected more obviously than we have seen in our experiments, where, we suspect, the TMS-induced disruption of state estimate was brief and may have largely recovered by the end of the reaching action. The consequences of experimentally delaying visual feedback, which effectively makes the state estimate inaccurate with respect to actual feedback, are similar to that of cerebellar inactivation [2,46]. Delayed state estimation would therefore explain loss of coordination and ataxia.

The state estimation process is likely to be iterative [8], with the current state being updated by an optimised weighting of afferent proprioceptive and visual information and by efferent motor commands. An iterative calculation is optimal, because previous state estimates, even if inaccurate, provide an additional source of information to be used in the new estimate. This is particularly true for physiological systems in which the state cannot change instantly; there must be a strong correlation between previous state and new states. We cannot tell from the present results to whether the 138 ms interval reflects “freezing” of the state estimate, until the cerebellum generates a new estimate, or whether it reflects the fall-back use of out-of-date proprioceptive and visual information. Possible methods to address this question would involve using longer trains of TMS, to stretch the time that it was perturbed, or to use repetitive TMS to induce a temporary “virtual lesion”, coupled with adaptation to delayed visual feedback.

However another important issue is whether the state estimate is localized entirely within the cerebellum or is distributed across this and other areas. One obvious candidate, given its well-documented role in spatial representations, is the posterior parietal cortex [47–49]. It has also been proposed as a locus of the state estimate [16,17,50] and has been recently implicated in the sense of agency and the mental representation of [51,52]; agency is also dependent on forward modelling [53]. Our data argue against the possibility that the state estimations are generated exclusively by the parietal cortex, because of the disruption caused by cerebellar TMS, but it seems plausible that both areas are involved. One possibility is that the parietal cortex maintains a body representation or body schema [50,54] that is updated during movements [16]. We would argue that this update is calculated by the cerebellum.

Anatomical connections to the cerebellum are consistent with this, as it receives powerful projections from posterior parietal cortex [55], which may hold a representation of the current state estimate [16], as well as from cortical motor areas, sending an efferent copy of descending commands [56]. It also receives visual and proprioceptive afferents, although in our task, visual feedback was blocked during the action. The output of the cerebellar processing, which we propose constitutes an estimation of the change in the motor state caused by the efferent signals, may then return to posterior parietal cortex [57–60] to update its representation, or be directed to motor areas to contribute to the control of the actions [56]. In this framework, one might expect that TMS of posterior parietal cortex would also disrupt reach-to-target actions in this task. Targeting the superior parietal cortex using the P3 electroencephalogram electrode coordinates did not cause significant effects. However, this negative result should be taken with caution, because we may have missed a critical locus within the posterior parietal cortex (PPC) responsible for maintaining the state representation.

The PPC also contributes to the representation of target positions [61], and hence it is important to distinguish loss-of-state estimation from errors in localizing the remembered target. This might be a result of TMS-perturbed input to the PPC, or it may be possibly due to a cerebellar role in target localization. However, we saw no evidence for mislocalization of the target in any of our conditions. In particular, we found direction-specific changes in initial movement direction with cerebellar TMS that were uncorrelated with changes in end position of the reach-to-target movement. Moreover, these effects were significantly different from the errors caused by cerebellar TMS when the hand was initially stationary, although one might expect any effect of target mislocalization to be common across all these three conditions. There is limited published evidence of a role for the cerebellum in localization of a visual target [62], and several opposing results [63–65]. Thus, it seems unlikely that the changes in direction were a sign of movements planned towards a perturbed position,

However, comparing the TMS data from the stationary start condition to the two active movement conditions does raise another concern, because it is well known that the TMS thresholds in motor cortex are lower during active movement than during rest. We did not test thresholds for activation over the cerebellum; however, we note that the arm was not at rest in this static position but was actively held in the air just above the start key; this is a motor task in which the cerebellum is actively engaged [66–68]. Others have used single-shock stimulation levels of 55% with the same double-cone coil and have seen brief changes in excitability of the contralateral motor cortex that are consistent with activation of the cerebellar cortex [69]; we have seen the same effects on muscle-evoked potential (MEP) amplitude (unpublished results) using triplets of 20-Hz pulses as used in the present experiments, even at stimulation levels of as low as 35% and with the arm genuinely at rest. So although it is possible that our TMS protocol was less effective during the stationary condition, we do not think this likely to have influenced these results.

### Control Experiments and Other Considerations

The TMS pulse train is expected to lead to a temporary disruption of the neural processing in underlying target tissue. We targeted the hand area of the ipsilateral cerebellar cortex at a site at which we have previously caused disruption of visually guided action [70], and at which TMS is known to affect cerebellar–cerebral projections leading to measurable changes in motor cortical excitation in the contralateral hand area [69]. One important control condition was therefore to test the same TMS protocol applied directly to the contralateral motor cortex, to rule out indirect effects of the cerebellar stimulation at this remote site. Motor cortical TMS did raise terminal errors lead to some directional deviation of the reaching movement, but both of these effects were of a significantly smaller magnitude than those seen after cerebellar stimulation (Figures 2 and 3).

At the same time, the cerebellar TMS stimulation caused a noticeable change in movement kinematics, with a significant reduction in movement onset latency and an increase in peak velocity. Similar but weaker effects were also generated by stimulation over the neck, at a site 3 cm below the cerebellar stimulation site. We reproduced the cerebellar effect on reaction times using a startling stimulus that did not involve functional TMS, either by using one wing of the double-cone coil placed over the participants' right ear to induce the noise and possible auditory nerve stimulation caused by the TMS stimulation over the cerebellum or neck, or by playing loud white noise bursts through headphones without any active TMS. Thus TMS aimed at the lateral cerebellum can startle the participant and lead to changes in the velocity profile of the movement, regardless of its effect on the cerebellum. However, even though these control conditions could induce a similar magnitude shift in reaction time and increase in peak velocity, they induced neither the terminal errors nor the initial directional errors that were caused by cerebellar TMS. This confirms that the initial directional deviation and the final positional errors were not a result of the startle effect. Furthermore, we also tested cerebellar stimulation from a static starting position, a condition that minimizes the need for a dynamic update of the state estimation. Again, we saw change in movement onset and velocity attributable to startle, but we saw no directional deviation or terminal error.

It is also possible that TMS applied over the lateral cerebellum could cause movement errors due to direct stimulation of muscles in the neck, which might lead to shoulder or upper arm deviation or cause arm movement by stimulation of the brachial plexus [69]. We discounted both possibilities by testing TMS stimulation over the neck at a site more likely to activate the brachial plexus, and that stimulation generated visible twitches in the neck muscles but without inducing the directional or terminal errors. TMS at the level used (45% of machine output) is unlikely to cause cortico-spinal stimulation [69,71]. Another control involved measuring the total deviation of the hand that was held static over the start position, without any active reaching task, during TMS of the cerebellum. This would expose any involuntary hand motion induced either by the TMS, including activation of the cortico-spinal collaterals [71], or by its startling effect. On TMS trials (three participants), the index finger was briefly deviated laterally by less than 5 mm, and within 200 ms had returned to within 1 mm of its initial position, within the normal reaction time period (266 ms). Hence our results are unlikely to be due to TMS-induced peripheral effects.

A final methodological consideration is that the significantly reduced reaction times seen after cerebellar TMS mean that the reach-to-target starts from a position near to the mean hand position at which the angular difference between TMS and non-TMS trials is equal (Figure 3A). In other words, one could argue that the TMS has merely shifted the mean start position leftwards in accordance with the reduction in reaction time, and has not affected the internal state estimate of the hand. However, the interval of 138 ms estimated from comparing directional errors on TMS versus non-TMS trials is significantly greater than the change in reaction time (93 ms). Furthermore, reduction in reaction time alone should not necessarily cause a directional error. If the reach-to-target movements on TMS trials were planned using an accurate state estimate, then their initial direction should be towards the target, despite their reduced latency. This result was clearly seen for the startle trials (Figure 3B), in which the reaction times were advanced by 85 ms, but the initial direction was unchanged. Thus the startling stimulus does not affect the movement direction, whereas cerebellar TMS does, and this dissociates the effects of reduction in reaction time from the loss of state estimation of the hand.

In conclusion, we suggest that these results indicate that the lateral cerebellum is responsible for estimating the true state of the peripheral motor system over a short time interval. We assume this estimation is based on forward modelling of the expected consequences of outgoing motor commands and that the updated estimation is sent from the cerebellum to cerebral areas responsible for planning and controlling the reaching action. These experiments do not tell us how the cerebellum generates these signals, whether the TMS protocol has any influence on cerebellar learning, or whether state estimates are topographically organized in the cerebellar cortex or nuclei. Experiments using methods with finer spatial resolution than TMS will be needed to address these important questions.

## Materials and Methods

### Participants.

Forty-five right-handed participants (age range 22–48 y, 13 male) received TMS, after providing informed written consent, and with approval from the Central Office for Research Ethics Committees. Two of these were authors of this article: RCM and LODC. Seven participants (age range 22–50, 6 male) were tested with auditory stimulation, including the authors RCM and JS.

### Tasks.

Participants sat at a table with their head supported by a chin rest and wearing Plato LCD goggles (Translucent Tech). A TMS coil was held in position using a Magic-Arm (Adaptivation). The position of the right index finger was recorded using a Polhemus Fastrak at 120 Hz. Trials were timed by a computer running under DOS. Each trial began with the index finger depressing a start key on the table top in front of the right shoulder. Cued by a set of three rising tones at 500-ms intervals, the participant was required to release the start key on the third tone, and to begin to move the right hand towards the right side. The Plato goggles were switched to opaque as soon as the start key was released. Early or late release of the key led to the trial being aborted. At a uniform random time 500–1,500 ms after the key was released, the onset of a fourth continuous tone cued the participant to make a rapid forwards and upwards reach to place the index finger on the position of a virtual target image, reflecting a 1-cm target in a mirror. The target was approximately 28 cm above and 15 cm in front of the start key. One second after this final go cue, the Plato goggles were switched to transparent, allowing terminal vision of the static finger and virtual target. The subject then returned to the start key at their own pace.

On each session, participants were given 60 practice trials, on a random 50% of which a series of three TMS clicks were heard, at 50, 100, and 150 ms after the onset of the go cue. During training, the coil was held about 1 m from the head; training data were only recorded for 18 participants. Immediately after the practise, the TMS coil was positioned against the scalp, and another 60 trials collected, with active TMS on half the trials.

Nine participants were also tested in a condition is which the start key was moved 20 cm laterally, to the average position at which the reach to the target started (Figure 1B). Subjects were instructed to lift the index finger off the start key but to remain stationary until the go cue signalled the rapid reach to the target. All other aspects of the task remained the same.

### TMS.

Repetitive stimulation was delivered as three biphasic pulses triggered at 20 Hz (50 ms) by the experimental control computer, at 45% of machine output, using a Magstim Rapid (Magstim Co.). For stimulation of the lateral cerebellum (*n* = 32), a 90-mm radius double-cone coil was centred 3 cm lateral and 1 cm below the inion [69,70]. In this position, one wing of the coil normally overlaps the participant's right ear. Ear plugs were provided.

To test the effects of TMS noise and its possible stimulation of the right ear, the orientation of the double cone coil was reversed in four participants so that one coil surrounded the right ear while the other was approximately normal to the scalp. Biphasic stimulation was set at 45% of the machine output. These data were combined with that of a group of seven participants in which the TMS trigger pulses were used to trigger brief white noise bursts (100 dB, 20-ms duration), played through binaural headphones. This white noise was sufficiently loud to evoke observable reflexive blinks in all participants, while remaining within safety limits. Comparison of the data from the two groups (ear TMS versus auditory stimulation) revealed no significant differences, and the two datasets were combined.

For stimulation of the neck (*n* = 11) a flat, 70-mm radius figure-of-eight coil was used, with the coil centre 3 cm below the site used for cerebellar stimulation (3 cm lateral and 4 cm below the inion). Stimulator output was set at 45% of machine output.

For stimulation of the motor cortex (*n* = 20) the flat, 70-mm radius figure-of-eight coil was positioned at a site where an observable twitch of the right first dorsal interoseus muscle was seen. Stimulator output was set at the resting threshold. For posterior parietal cortex, all participants (*n* = 12) were also tested with M1 stimulation and the same stimulator intensity was used. The P3 electrode position was measured using standard landmarks.

### Data analysis.

Index finger trajectories were analysed in Matlab version R2007a. A Polhemus Fastrak receiver was taped above the right index finger, and before the experiment began, each participant held the index finger stationary in the position of the virtual target, under full vision. The recorded marker position was then taken as the target position in all subsequent analysis, accounting for the 1–1.5 cm positional offset of the marker from the index finger pad. Finger position was recorded in three axes at 120 Hz; angular rotations of the hand that would invalidate this positional offset were minimal and estimated at less than 1 mm; the relative difference between TMS and non-TMS trials is less than 10% of this (0.1 mm).

### TMS artefacts.

TMS magnetic pulses can generate a significant one-sample (8 ms) artefact in the Polhemus motion tracking data, which uses magnetic field technology. These artefacts were detected in the first-differenced time series data and removed by interpolation across neighbouring data points.

To assess the impact of these artefacts, we recorded the apparent marker position of a static marker placed at the average start position of the reach-to-target movement, with the TMS coil placed in approximate similar position as when testing a participant. Artefact removal was successful and the apparent residual motion of the marker was under 0.1 cm or 1 cm/s. The duration of the artefact was also restricted mainly to within the typical reaction time, so any residual error did not affect analysis of the reach trajectory. Testing with the marker attached to a participant's finger held stationary while TMS was applied to the cerebellum (three participants, 20 trials each) showed that stimulation of the cerebellum caused minor finger motion that was recovered within 200 ms of TMS termination.

The cleaned positional data were then low-pass filtered (8th order zero phase 7.5 Hz Butterworth filter). Low-pass filtered index finger trajectories were differentiated to velocity, and the tangential speed was averaged across all TMS (*n* = 30) and non-TMS trials (*n* = 30) per subject for each condition. The subject mean velocities were then averaged across the subject group.

Mean speed and jerk (second derivative of speed) profiles were examined before and after the removal of the TMS-artefacts in the Polhemus data to confirm that the artefact removal was effective. All trials (TMS and non-TMS) were processed and filtered identically.

### Detection of terminal and directional error.

The following steps were taken for analysis of each trial. First, the time-point of maximum velocity was detected between the go cue and the end of recording (open circles, Figure 1B and 1C). Termination of the reach-to-target was then taken as the time point at which movement velocity fell below 5% of the maximum (filled circles and trial number, Figure 1B and 1C).

Reach-to-target movement onset was detected as the point of maximum curvature between the go cue and the point of maximum velocity (asterisks, Figure 1B and 1C). To find this, each trajectory from go cue to termination was spatially re-sampled to 100 uniformly spaced points, the rate of change of these spatial positions then recorded as curvature, and the maximum curvature spatial position found. The time point of original data sample the within the original time series closest to this spatial position was then recorded as the time point at which the reach movement initiated.

End point error was measured as the Cartesian distance of the finger from the target. Directional errors were measured as the azimuth or elevation differenced between lines joining the start point and target, versus the start point and maximum velocity point for each trial. Mean angular differences in azimuth and elevation between all TMS (*n* = 30) and all non-TMS trials (*n* = 30) were calculated per subject for each experimental session.

### Estimation of predictive interval.

To estimate the positional offset that corresponded to the directional error measured, the angle between the start position and the position of maximum velocity was calculated for the mean trajectory of all non-TMS trials (*n* = 30) for each subject in each condition. Trajectories were spatially re-sampled before averaging (e.g., Figure 1D and 1E). Then, by iteratively recalculating this angle for each data position before the start position, we found the first position at which the angular difference exceeded the mean angular difference between TMS and non-TMS trials (black dots, Figure 3). The distance along the mean trajectory between this position and the start position was found. To estimate the time interval that corresponded to this spatial offset, the mean velocity of the hand was found at cue onset, for each subject. Dividing the estimated offset, subject-by-subject by the mean velocity, we estimated the time interval of the state estimation.

**Figure 7:**
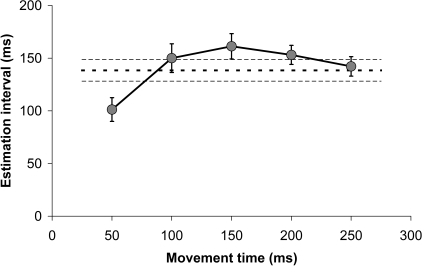
Sensitivity of the Calculation of Estimation Interval on the Reference Points Chosen to Measure Movement Deviation The five data points are the mean estimation interval (± SEM, *n* = 32) calculated at fixed times after movement onset. The dashed line is the mean estimate (±1 SEM) calculated from the point of maximum velocity in each trial.

To test the sensitivity of this analysis to the arbitrary choice of the point of maximum velocity as a reference position, we repeated the above analysis choosing instead five time points in 50-ms steps from 50–250 ms after the start of the reach-to-target movement; the maximum velocity was normally reached at about 250 ms (Figure 6). The first 50 ms estimate was significantly different from all others (Figure 7, Bonferroni adjusted *p* < 0.001, paired *t*-tests); the other four estimates did not significantly differ (Bonferroni adjusted *p* > 0.3), and did not differ from the estimate based on the maximal velocity (dashed lines, Figure 7). Hence, we are satisfied that the results we present here are largely insensitive to the precise reference point chosen within the reach-to-target trajectory at which we assess movement direction, and may somewhat underestimate the magnitude of the time interval. Only when this reference position is close to the initiation point of the movement (50 ms, or 6 data sample) was the estimate lower than that calculate from the point of maximum velocity. Figure 7 also makes clear that the highest estimate of the interval (161 ± 11.9 ms) was found using a reference point 150 ms into the movement, and this may then indicate that this (150–160 ms) is indeed the best estimate; additional experimental work will be required to get independence evidence of this, however.

## Abbreviations

CNS: central nervous system
PPC: posterior parietal cortex
TMS: transcranial magnetic stimulation
